# Experiences in the Management of Type A Aortic Dissection Following Cardiovascular Interventions: A Case Series Report

**DOI:** 10.3390/jcm15207901

**Published:** 2026-10-13

**Authors:** Lile He, Zhiwei Zhang, Tao Tang

**Affiliations:** Department of Cardiovascular Surgery, The Second Xiangya Hospital, Central South University, Changsha 410011, China

**Keywords:** cardiovascular intervention, aortic dissection, iatrogenic aortic dissection, TAVR, TEER, Bentall procedure, case report

## Abstract

**Background:** Cardiovascular interventions such as transcatheter aortic valve replacement (TAVR) and transcatheter edge-to-edge repair (TEER) have become standard options for high-risk patients with valvular heart disease. However, iatrogenic type A aortic dissection (AAD) remains a rare but life-threatening complication, and its optimal management is controversial. **Case presentation:** We present four patients who developed AAD following cardiovascular interventions at our institution between 2020 and 2024, including three after TAVR and one after TEER. Three patients underwent the Bentall procedure (each requiring removal of the pre-existing transcatheter valve, and one with concomitant coronary artery bypass grafting), and one underwent aortic sinus repair with ascending aorta and total arch replacement using a frozen elephant trunk, together with removal of the previously implanted TEER clip and mitral valve replacement. One patient recovered and was discharged home, another was transferred back to the cardiology department and was discharged home with no sequelae, and two left the hospital against medical advice while still in critical condition, both of whom subsequently died—one 2 days after discharge and the other immediately upon arriving home. **Conclusions:** Our experience indicates that patients with cardiovascular intervention-related AAD are at particularly high risk owing to pre-existing cardiac dysfunction and deterioration of other organ functions. In such patients, emergency surgical rescue can be life-saving, and the Bentall procedure proved feasible in this selected cohort; however, surgery in this setting is more complex than in routine cases. Careful evaluation, individualized surgical planning, and meticulous perioperative management are essential to improve outcomes.

## 1. Introduction

Catheter-based cardiovascular interventions have expanded at a remarkable pace over the past two decades and now constitute a cornerstone in the management of structural heart disease [1]. Their rapid adoption mirrors the persistent burden of cardiovascular disorders, which remain the leading cause of mortality worldwide [2].

Transcatheter aortic valve replacement (TAVR) is now firmly established as an alternative to surgical aortic valve replacement for symptomatic severe aortic stenosis, and its indications have progressively widened from patients at high or prohibitive surgical risk to those at intermediate and low risk [3,4]. Transcatheter edge-to-edge repair (TEER) has followed a comparable trajectory for degenerative mitral regurgitation [5]. Because both procedures are delivered through peripheral vascular access, they obviate sternotomy and cardiopulmonary bypass, shorten convalescence, and extend definitive valve therapy to patients who would previously have been managed conservatively [6].

Alongside this widening indication profile, iatrogenic complications remain an inherent hazard of intravascular instrumentation, and aortic dissection is among the most feared of them, because it converts an elective minimally invasive procedure into a life-threatening surgical emergency [6,7]. Refinements in device design, delivery technique, and patient selection have lowered—but not abolished—the incidence of this event [8,9]. Once the dissection involves the ascending aorta, malperfusion and rupture may supervene within hours, and the window for successful rescue is narrow [10].

In this report, we describe four patients who developed acute type A aortic dissection (AAD) during or after TAVR or TEER at our institution between 2020 and 2024, analyze the timing, anatomy, and management of the complication in each case, and summarize the practical lessons drawn from this experience, with the hope of assisting teams confronted with this rare but catastrophic scenario.

## 2. Materials and Methods

### 2.1. Study Design

This retrospective case series was conducted at the Department of Cardiovascular Surgery, The Second Xiangya Hospital, Central South University, China. Between 2020 and 2024, patients who sustained an acute type A aortic injury during, or immediately after, a transcatheter structural intervention (TAVR or TEER) and were managed by our cardiovascular surgical service were identified from institutional surgical and procedural records; four patients were identified and are reported here; to the best of our knowledge, these four patients represent all individuals with intervention-related type A aortic injury who were referred to our surgical service during the study period. An injury was classified as procedure-related when it occurred during the index intervention or within 24 h afterwards and was confirmed by intraoperative findings or imaging. For each patient, we collected baseline demographic, clinical, and echocardiographic characteristics; procedural details (access route, device type and size, timing of injury recognition); operative findings (entry-tear location, extent of injury, details of the repair); and in-hospital outcomes. Follow-up information was retrieved from outpatient records where available. Because only patients who reached our surgical service are included, this series is descriptive and is not intended to estimate the incidence of the complication. The study was approved by the Medical Ethics Committee of the Second Xiangya Hospital, Central South University (No. LYEC2024-K0168), and written informed consent for publication was obtained from all patients or their legally authorized representatives (see the Ethics Statement).

### 2.2. Case Presentations

**Figure 1:**
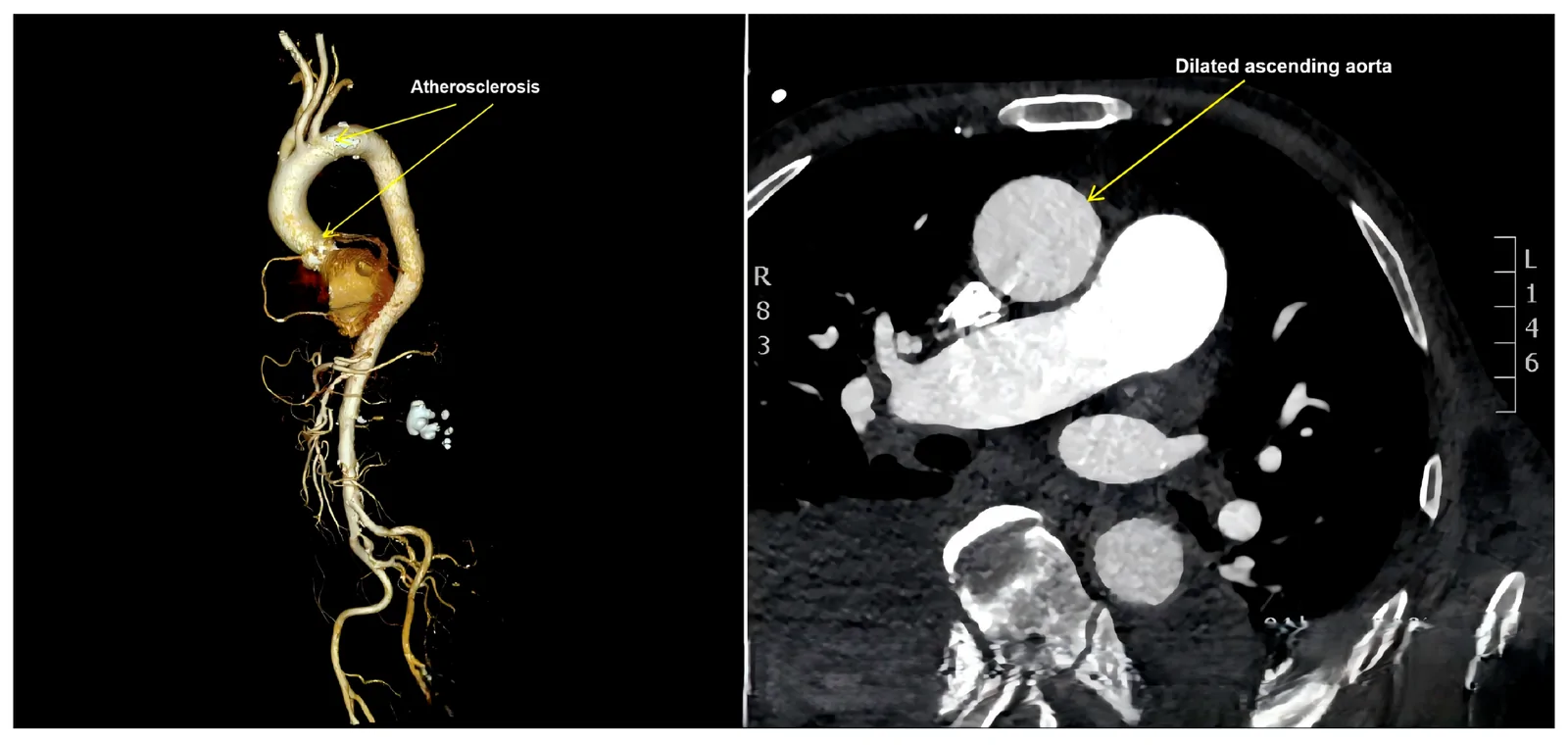
Preoperative CTA of patient 1. The CTA scan showed heavy atherosclerosis of the aorta in patient 1.

**Figure 2:**
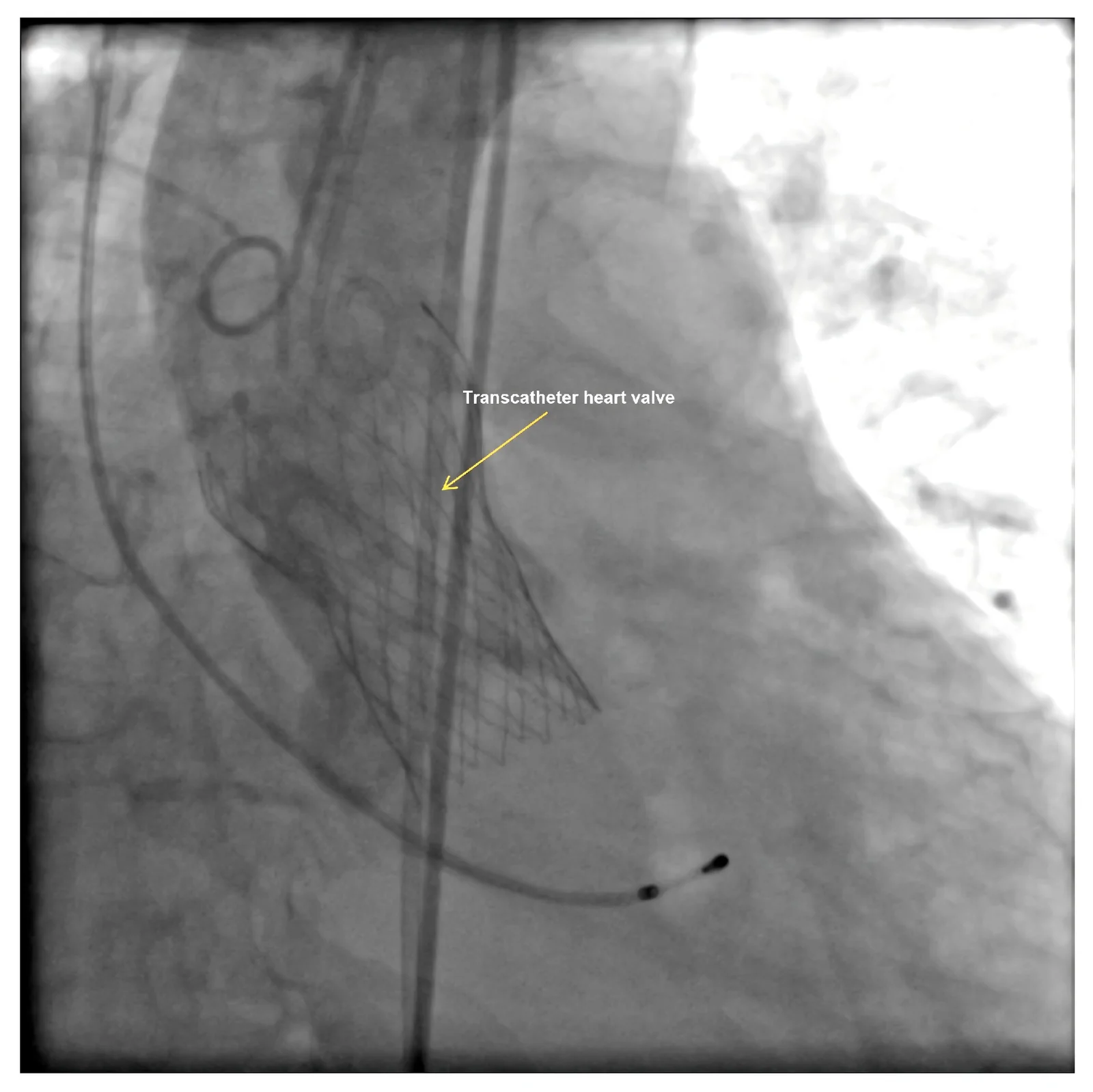
Fluoroscopic image of patient 1 during TAVR. The transcatheter heart valve was deployed across the aortic annulus. Immediately after deployment, the patient developed hemodynamic collapse, and echocardiography revealed rapidly enlarging pericardial effusion suggestive of iatrogenic type A aortic dissection.

The individual baseline and procedural characteristics of the four patients are summarized in Table 1. Patient 1, an 83-year-old man with decompensated severe calcific aortic stenosis, chronic kidney disease, hypertension, and coronary artery disease on long-term aspirin plus clopidogrel, was admitted with refractory heart failure (New York Heart Association class IV). Echocardiography showed LV 57 mm, LA 50 mm, RV 34 mm, RA 39 mm, ascending aorta 34 mm, and an ejection fraction of 28%, with diffuse hypokinesis, mild mitral and tricuspid regurgitation, and a small pericardial effusion; N-terminal pro-B-type natriuretic peptide (NT-proBNP) exceeded 35,000 pg/mL. Computed tomographic angiography (CTA) confirmed heavy atherosclerosis of the aorta (Figure 1). After heart-team evaluation—advanced age, poor cardiac function, chronic kidney disease, and prohibitive surgical risk—the decision was made to proceed with transfemoral TAVR. During the procedure, shortly after valve deployment, the blood pressure fell abruptly and echocardiography showed a rapidly enlarging pericardial effusion (Figure 2); an intervention-related aortic injury was suspected, and the patient was transferred for emergency surgery the same night.

**Figure 3:**
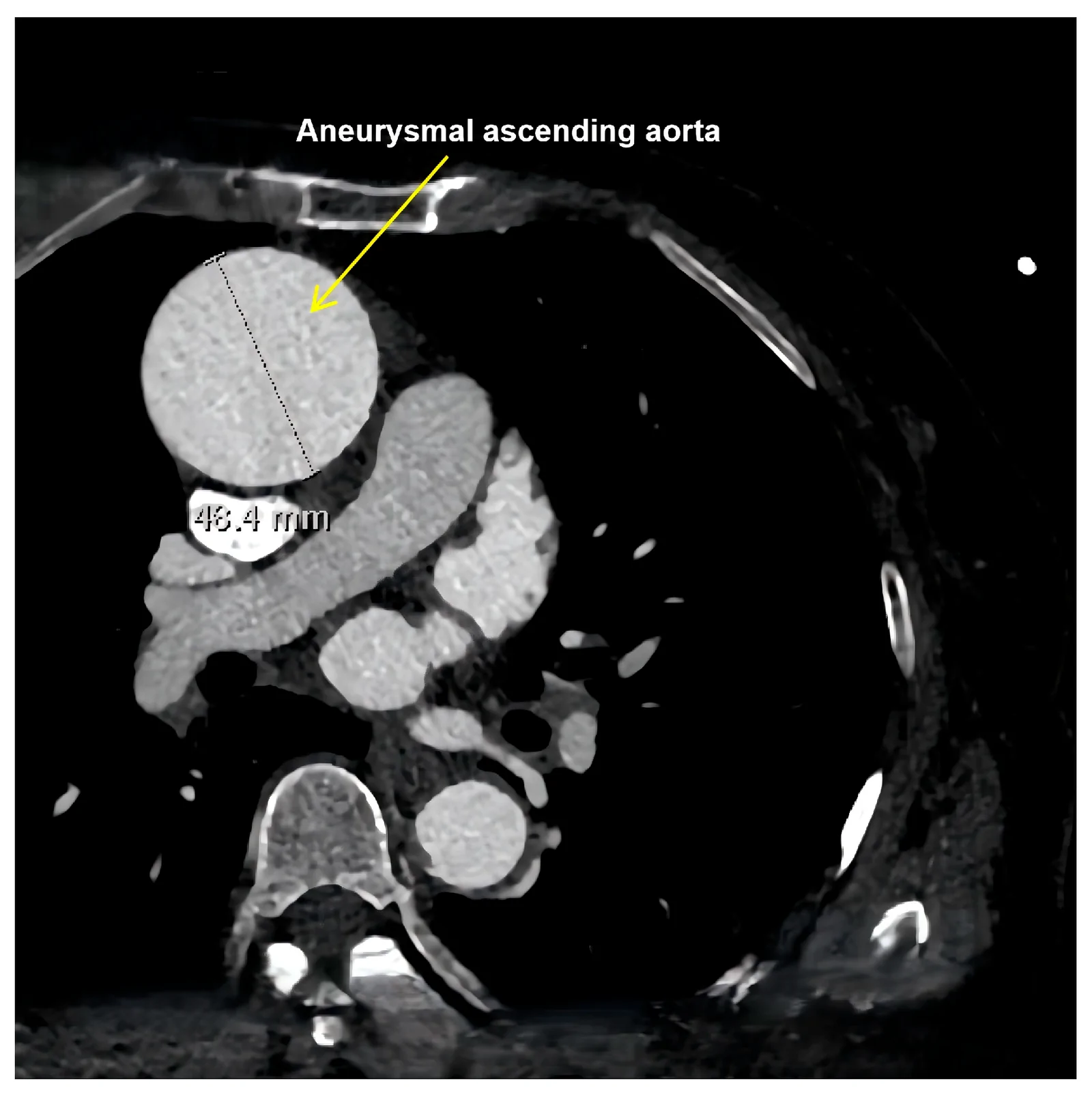
Preoperative CTA of patient 2. CTA scan showed that patient 2 had a dilatation of the ascending aorta.

**Figure 4:**
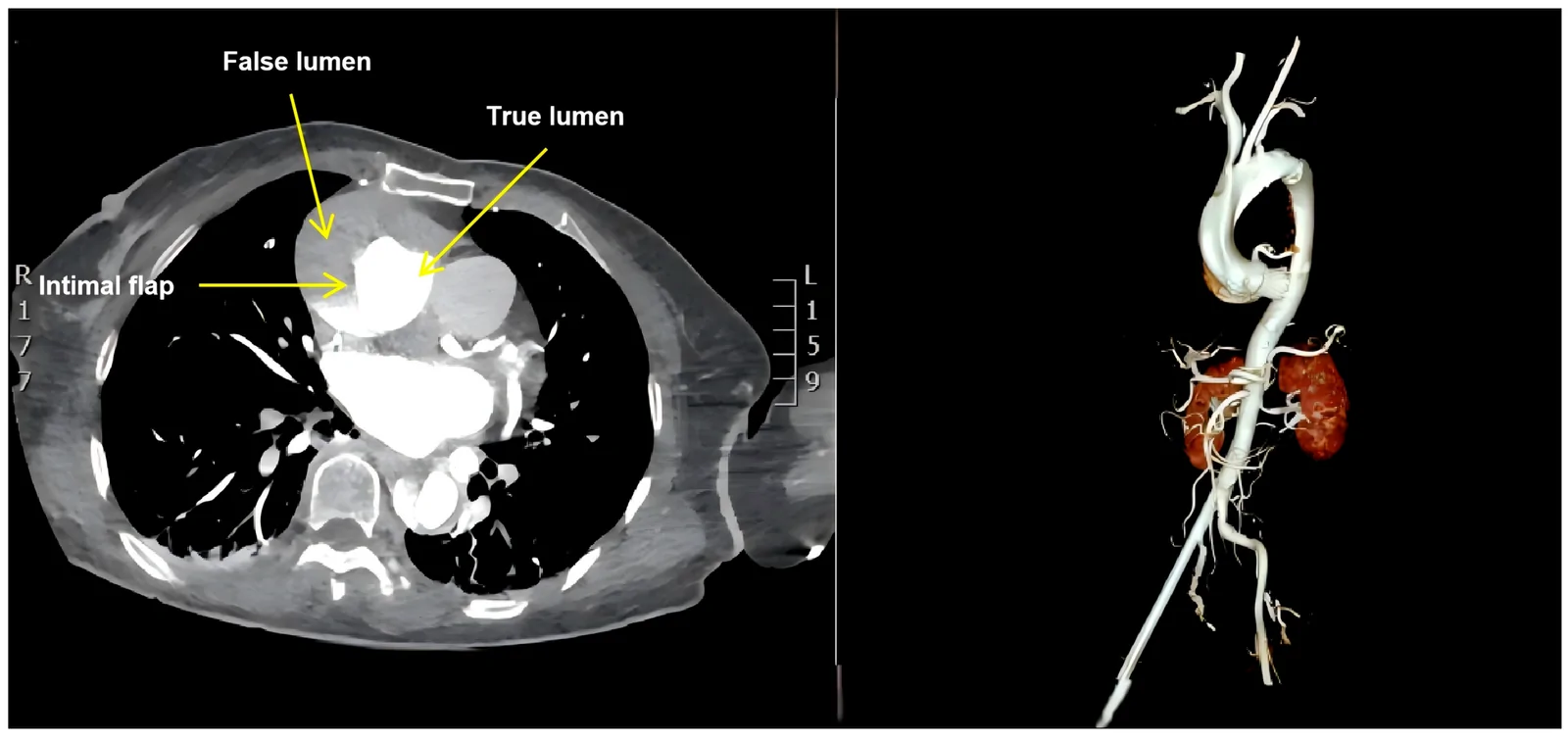
CTA after TAVR procedure of patient 2. An immediate CTA scan was performed and the formation of AAD was confirmed.

Patient 2, a 77-year-old woman with a bicuspid aortic valve, severe aortic stenosis, hypertension, and an ascending aortic aneurysm of 48 mm (Figure 3), with preserved left ventricular function (LVEF 56%) and an NT-proBNP of 207 pg/mL, was admitted for TAVR. The procedure was performed through a right femoral arterial access (20-F sheath) with an additional 6-F sheath in the left femoral artery. After crossing the stenotic valve, a 26-mm Medtronic (Minneapolis, MN, USA) transcatheter valve was deployed first; because anchoring was suboptimal, it was replaced with a 29-mm valve, which achieved a stable position with both coronary arteries clearly visualized on aortography and no paravalvular leakage. However, intraoperative transesophageal echocardiography (TEE) unexpectedly demonstrated intimal detachment of the ascending aorta. The patient remained hemodynamically stable, and an immediate CTA scan confirmed acute type A aortic dissection (Figure 4); she was transferred for emergency surgery later the same day.

**Figure 5:**
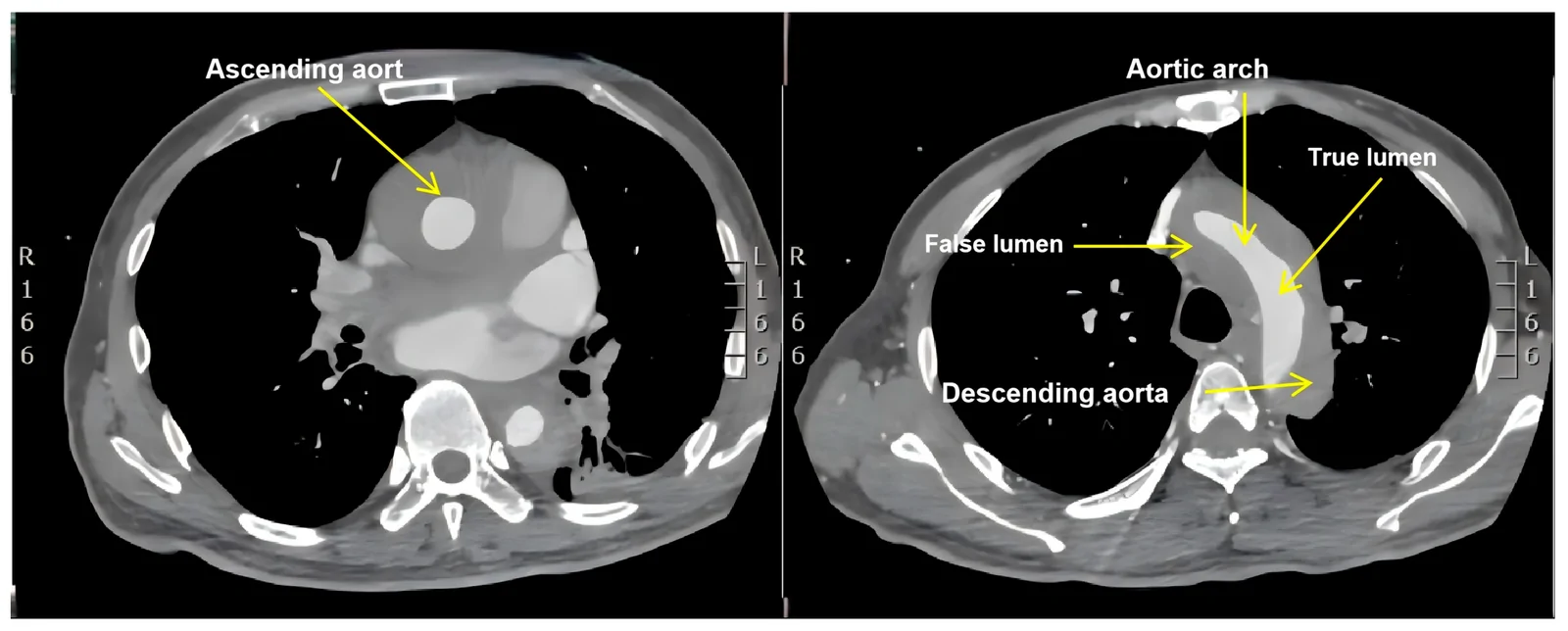
CTA after TEER procedure of patient 3. The CTA scan confirmed the formation of AAD.

Patient 3, a 58-year-old man with severe mitral regurgitation caused by prolapse of the P2 and P3 segments with ruptured chordae tendineae, severe tricuspid regurgitation, and a pulmonary artery systolic pressure of 59 mmHg, was admitted for TEER. Echocardiography showed four-chamber enlargement (LV 71 mm, LA 61 mm, RV 40 mm, RA 40 mm) with an ejection fraction of 54% and mild degenerative aortic valve disease; the NT-proBNP level was 5113 pg/mL. One week earlier, two drug-eluting stents had been implanted in the left anterior descending artery, and dual antiplatelet therapy with aspirin plus clopidogrel was maintained. Because of the high surgical risk associated with severe biventricular valvular disease and pulmonary hypertension, he was enrolled in a clinical trial of TEER using an investigational edge-to-edge clip system (Valveclip-M, Newmed Medical, Shanghai, China). The procedure was performed via an 8-F right femoral venous access pre-closed with a Perclose ProGlide device (Abbott Vascular, Santa Clara, CA, USA), followed by transseptal puncture (the distance from the puncture site to the mitral annulus was 4.4 cm) and advancement of a 24-F guiding sheath; heparin (7000 U) was administered with an activated clotting time of 315 s. A single clip was implanted to grasp the A3-P3 segments, but residual mitral regurgitation remained grade 3+. During clip manipulation, TEE suddenly revealed widening of the ascending aorta suspicious for an intramural hematoma; although blood pressure was promptly controlled with nitroglycerin, repeat TEE showed progression, and the procedure was terminated immediately. Emergency CTA confirmed an acute type A aortic dissection (Figure 5). The patient remained hemodynamically stable throughout.

Patient 4, a 72-year-old man with a bicuspid aortic valve (type 0) and severe calcific aortic stenosis, was admitted with decompensated heart failure, hypotension, and bilateral pleural effusion. The ejection fraction was 30% and the NT-proBNP level exceeded 35,000 pg/mL, indicating significant cardiac dysfunction. During the TAVR procedure, circulatory collapse occurred shortly after valve deployment; angiography suggested pericardial tamponade, and an intervention-related aortic injury with acute type A dissection was suspected. The patient was transferred directly to the operating room for emergency surgery on the same night.

### 2.3. Treatments

**Figure 6:**
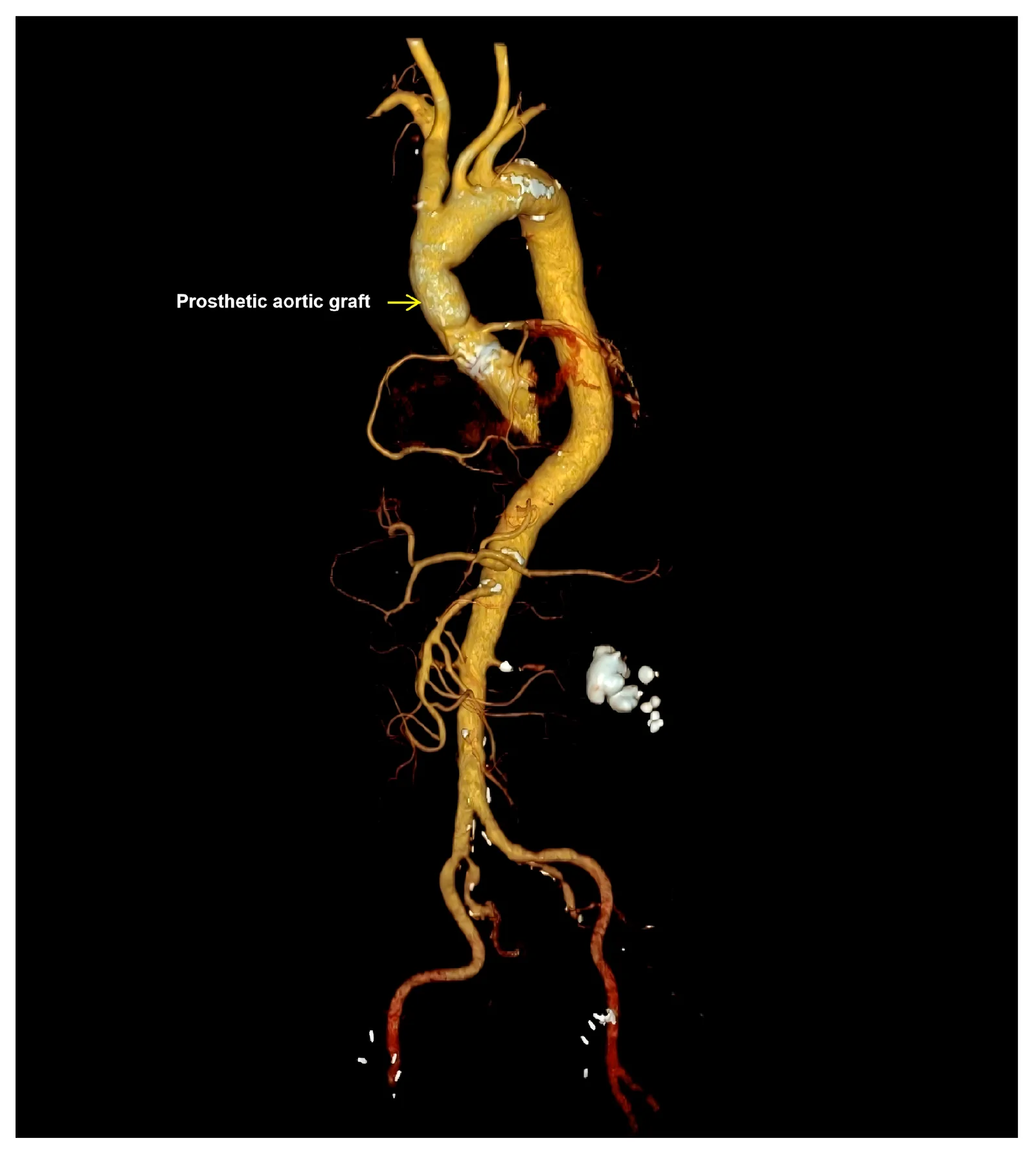
Post-operative CTA of patient 1. Postoperative CTA demonstrated the typical changes following the Bentall procedure, with unobstructed blood flow and no evidence of leakage in the aortic prosthetic graft.

Patient 1 underwent emergency surgery through a median sternotomy on the night of the TAVR procedure (Figure 6). Arterial cannulation was established via the left femoral artery with venous drainage from the right atrium; the cardiopulmonary bypass time was 346 min. Intraoperatively, an intimal tear approximately 3 mm in diameter was identified just below the left coronary ostium near the left-noncoronary commissure, with intramural hematoma involving the noncoronary and left coronary sinuses and extending into the ascending aorta. The transcatheter valve was explanted, and the aortic root and ascending aorta were replaced with a 21-mm mechanical valved conduit within a 26-mm vascular graft (Bentall procedure).

**Figure 7:**
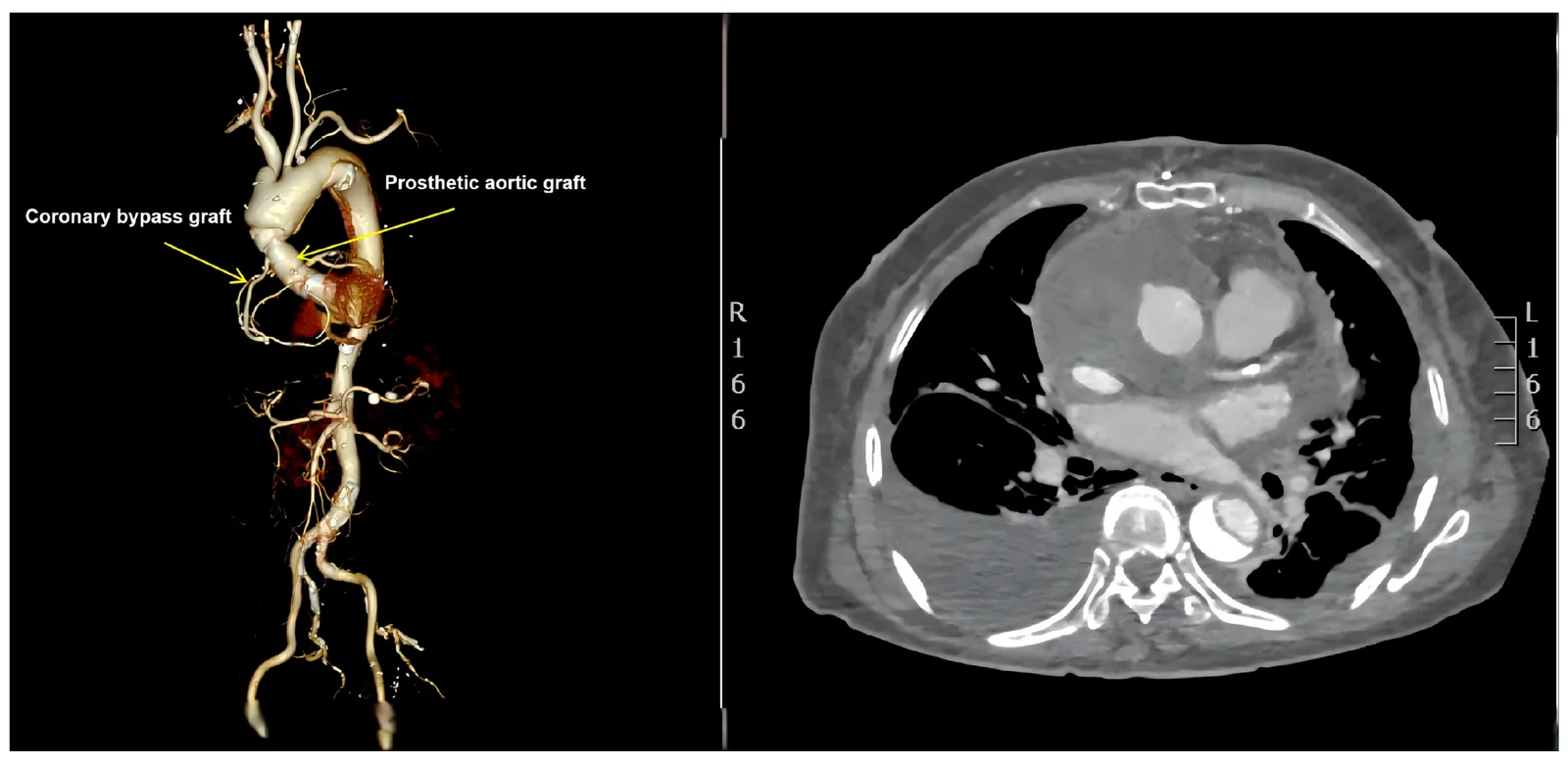
Post-operative CTA of patient 2. Postoperative CTA demonstrated the typical changes following the Bentall procedure with concomitant coronary artery bypass grafting, with unobstructed blood flow and no evidence of leakage in the aortic prosthetic graft; the residual arch intramural hematoma was managed conservatively.

Patient 2 was taken to the operating room later the same day. Through a median sternotomy with right axillary arterial cannulation, the previously deployed transcatheter valve was excised, and an intimal tear approximately 2 cm long was found as a transverse laceration within the noncoronary sinus. Because the heavily calcified right coronary ostium could not be preserved, it was closed during root replacement, and two saphenous vein grafts were anastomosed to the right coronary artery and the posterior descending artery. The Bentall procedure completed the repair (Figure 7).

Patient 3 remained hemodynamically stable after the diagnosis was established, and surgery was performed on the following day. The diagnosis-to-surgery interval mainly reflected the time needed for the family to deliberate on and provide informed consent for a high-risk open-heart procedure, given severe biventricular valvular disease and pulmonary hypertension; while strict blood-pressure control and close clinical surveillance were maintained, the surgical team, perfusion staff, and blood products were mobilized. After consent was obtained, the operation was performed through a median sternotomy with right axillary arterial cannulation (cardiopulmonary bypass 419 min, aortic cross-clamp 252 min, antegrade cerebral perfusion 25 min). Intraoperatively, extensive hematoma was found on the right ventricular surface and the interatrial septum, with a small perforation at the inferior margin of the fossa ovalis; the ascending aorta was thickened to approximately 4.5 cm, and the dissection involved the ascending aorta, the aortic arch, and the descending thoracic aorta with all three arch branches, accompanied by abundant thrombus in the false lumen. The procedure included aortic sinus repair, ascending aorta and total arch replacement with a frozen elephant-trunk stent graft (CRONUS (Lifetech Scientific, Shenzhen, China), 26 mm × 120 mm; Sun’s procedure) [11], removal of the previously implanted TEER clip, and mitral valve replacement with a 27-mm bioprosthesis.

**Figure 8:**
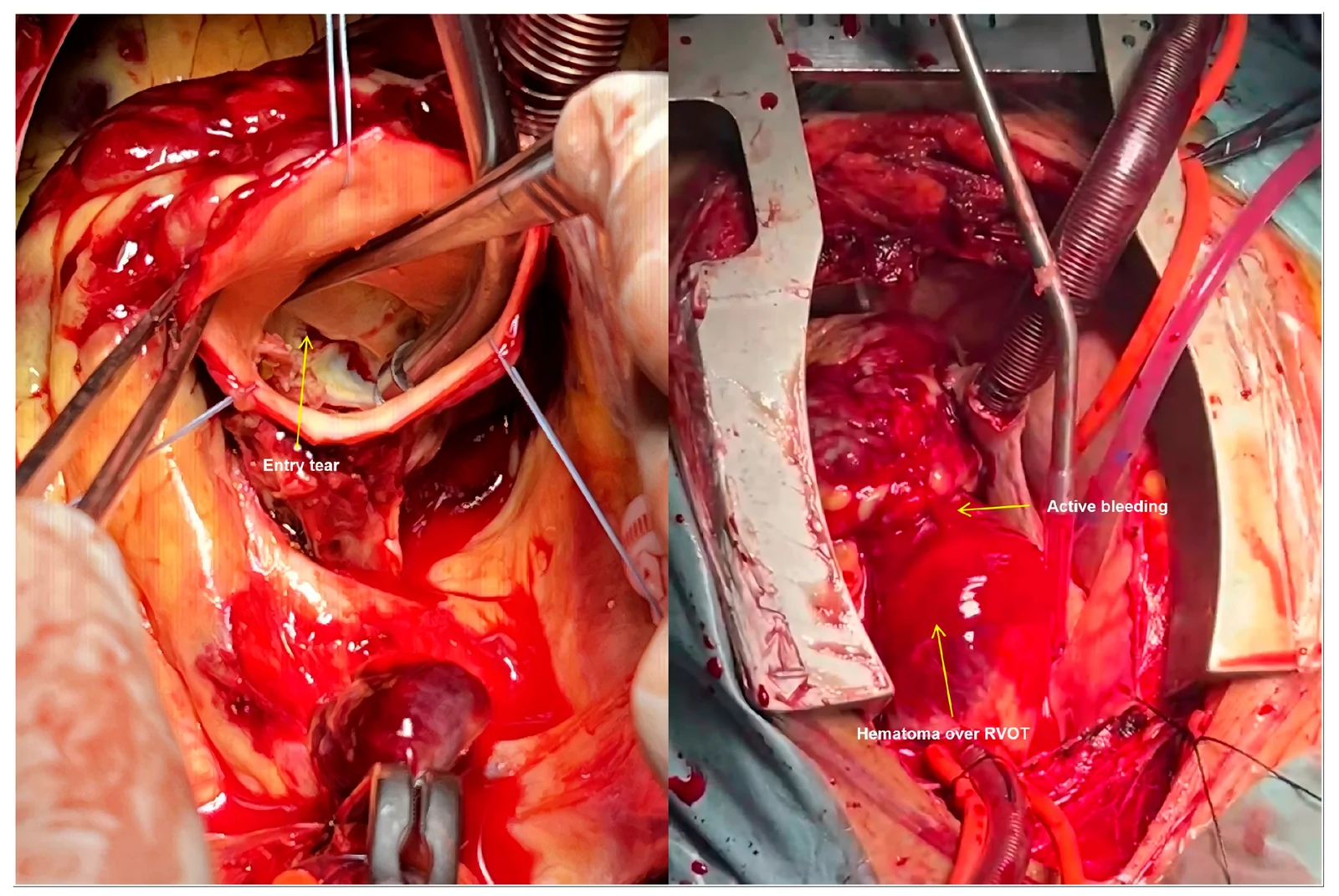
Intraoperative findings of patient 4. We observed significant pericardial effusion and hematoma formation on the right ventricular outflow tract, accompanied by active bleeding. There was also hematoma formation on the ascending aorta, and an intimal tear 2 mm in diameter was identified just below the right coronary ostium.

Patient 4 was transferred to the operating room at midnight for an emergency median sternotomy with right axillary arterial cannulation, which revealed massive hemopericardium and an actively bleeding hematoma on the surface of the right ventricular outflow tract, together with hematoma formation of the ascending aorta (Figure 8). An intimal tear 2 mm in diameter was identified just below the right coronary ostium, approximately 1 mm above the valve annulus. The transcatheter valve was explanted, and a Bentall procedure was performed with a 21-mm mechanical valved conduit within a 24-mm vascular graft.

## 3. Results

Two of the four patients (50%) died within 30 days of the index procedure; both deaths occurred after discharge against medical advice and are counted as early mortality of the acute episode. Patient 1 regained consciousness on the first postoperative day, but his recovery was complicated by sepsis with severe pneumonia, acute kidney injury requiring continuous renal replacement therapy, and gastrointestinal hemorrhage. Despite maximal intensive care, he remained critically ill and ventilator-dependent; on postoperative day 18, the family requested discharge against medical advice, and he died 2 days after discharge (postoperative day 20). Patient 2 recovered uneventfully from surgery; postoperative CTA showed a residual intramural hematoma involving the arch, which was managed conservatively because reoperation offered little benefit at considerable risk, and approximately 700 mL of pleural effusion was drained by closed thoracic drainage on postoperative day 14. She was discharged home in stable condition on postoperative day 16 with lifelong warfarin anticoagulation. Patient 3 suffered a witnessed cardiac arrest on the night of surgery, which was successfully resuscitated by cardiopulmonary resuscitation; his course was further complicated by anemia, infection, disturbance of consciousness, and hepatic and renal dysfunction. He was transferred back to the cardiology department for continued treatment on postoperative day 16 and was discharged home in stable condition with no sequelae on postoperative day 24 (28 March 2023). Patient 4 developed recurrent cardiac arrest with ventricular tachycardia shortly after surgery, which was resuscitated; however, he remained in refractory cardiogenic shock with pulmonary edema, copious pink frothy sputum, and persistent hypoxemia despite high-dose vasopressors (dopamine and noradrenaline). After the family declined mechanical circulatory support and signed a refusal of further treatment, he was discharged against medical advice on the day of surgery and kept on high-dose vasopressors during transport at the family’s wish that he pass away at home; he died immediately after arriving home. At follow-up, patient 2 remained alive and well at 31 months after surgery (September 2026); her clinical follow-up was unremarkable, and imaging follow-up (echocardiography at 6 months with normal prosthetic valve and ascending graft function, plus periodic computed tomography at a local hospital) has shown the residual arch hematoma unchanged since discharge. Patient 3 remains alive, self-caring, and asymptomatic at 42 months after surgery (September 2026); his clinical follow-up is unremarkable, but no imaging follow-up was performed after discharge.

## 4. Discussion

Since their clinical introduction, transcatheter valve therapies have provided a minimally invasive solution for severe valvular heart disease, above all in patients regarded as high-risk or inoperable. These procedures have improved prognosis and shortened recovery for a growing number of patients, yet they carry a distinctive complication profile of their own; although aortic dissection is among the rarest of these events, it is also among the most devastating.

### 4.1. Incidence and Mortality

Cribier and colleagues performed the first-in-human TAVR in France in 2002 [12]. Since then, iatrogenic aortic dissection has been recognized as a rare but catastrophic procedural complication, with an estimated incidence of 0.2–0.3% reported in TAVR series [13,14]; the aortic root is the segment most frequently involved, although the tubular ascending aorta can also be affected [15]. Even at this low rate, the steadily growing volume of transcatheter procedures performed worldwide means that a considerable number of patients encounter this life-threatening vascular injury each year [16]. The outcomes are sobering. Long-term survival has been documented after treatment of cardiovascular intervention-related AAD; in a contemporary review of post-TAVR aortic dissection, however, approximately 67% of affected patients died within 30 days [17]—a figure derived from larger cohorts that is soberingly consistent with the 50% (2 of 4) early mortality observed in the present small series. One analysis observed that patients sustaining AAD after TAVR are more often women aged 80 years or older in whom smaller transcatheter valves had been implanted [17]; no consistent predilection has emerged for balloon-expandable over self-expanding prostheses, or for smaller over larger valve sizes.

### 4.2. Risk Factors

Aortic dissection begins when an intimal tear allows blood to enter the aortic media, creating a false lumen and splitting the layers of the vessel wall. During cardiovascular interventions, several distinct mechanisms can initiate this process [18,19,20]. The first is direct mechanical trauma: guidewires, catheters, and bulky delivery systems are advanced and torqued repeatedly across the aortic arch and around the root, and the intima at these sites is especially susceptible to abrasion or perforation. The second is the radial force generated at valve deployment. Balloon-expandable and self-expanding prostheses alike exert outward pressure on the annulus and the adjacent aortic wall; an oversized prosthesis, an undersized annulus, or aggressive post-dilation can transmit stress sufficient to split a calcified wall. Third, the quality of the aortic substrate itself is decisive: heavy annular and leaflet calcification and extensive atherosclerotic plaque render the wall stiff and non-compliant, so that it tears rather than deforms under mechanical stress. Finally, pre-existing aortic pathology—aneurysm, connective tissue disorders such as Marfan or Ehlers–Danlos syndrome, or previous aortic surgery—weakens the structural integrity of the wall and lowers the threshold for dissection [15,18,19,21]. In most reported cases, therefore, intervention-related dissection reflects the convergence of an unfavorable aortic substrate with procedural mechanical stress rather than a single causative factor.

In our series, each of these mechanisms could be mapped to an individual patient (Table 2). In patients 1 and 4, the tears were located immediately below the left and right coronary ostia, respectively—precisely where the radial force of the prosthesis and balloon-valve interaction concentrates in heavily calcified roots. In patient 2, a bicuspid valve with a pre-existing 48 mm ascending aneurysm provided a well-recognized substrate for aortic injury, and the exchange of a 26 mm valve for a 29 mm prosthesis because of suboptimal anchoring plausibly increased the radial stress transmitted to the noncoronary sinus, where a transverse laceration of approximately 2 cm was found. The mechanism in patient 3 is the most instructive. Conventional TEER is performed entirely through a venous and transseptal route, and none of the instruments crosses the aortic valve or the aortic root; an ascending aortic injury during TEER is therefore particularly striking. At emergency surgery, an extensive hematoma of the right ventricular surface and the interatrial septum with a small perforation at the inferior margin of the fossa ovalis was documented, whereas an aortic entry tear was not identified. The intraoperative findings raise the possibility that the injury originated at the interatrial septum—most plausibly from the transseptal puncture or the manipulation of the 24-F guiding sheath—and then propagated through the periaortic tissue to the aortic root and ascending aorta, rather than resulting from direct endoluminal aortic trauma. However, because no aortic entry tear was identified intraoperatively, the direction of propagation and the causal link between the septal injury and the type A dissection remain hypothetical rather than established; in a severely enlarged right heart with recent coronary stenting on dual antiplatelet therapy, this constellation represents the most plausible, though unproven, explanation for this rare event.

### 4.3. Management Strategies

For spontaneous AAD, emergency surgical repair remains the reference treatment; in-hospital mortality among patients managed without surgery has been reported at 58%, compared with 26% among those treated surgically [22]. The published experience with intervention-related AAD includes open surgical repair [23,24], thoracic endovascular aortic repair (TEVAR) with covered stents [25], and bail-out endovascular maneuvers to restore perfusion of malperfused supra-aortic branches [20]. Endovascular treatment of the ascending segment, however, is technically demanding and far from standardized, because the anatomy of the aortic root and arch complicates graft landing and fixation; although a series using modified stent-grafts reported a 92% device success rate [26], these findings await validation in larger cohorts. It should also be recognized that most patients selected for TAVR or TEER are elderly, frail, and multi-morbid—precisely the profile in which reoperative open surgery carries prohibitive risk—even so, emergency surgery remains the reference treatment for acute type A aortic dissection, and non-operative management should be regarded only as an exceptional strategy—reserved for highly selected patients without a definite ascending intimal tear, pericardial effusion or tamponade, rupture, malperfusion, progression on serial imaging, or hemodynamic instability—rather than as an equivalent alternative [18,19,21]. Observations that medical therapy combined with timely operation can achieve favorable long-term outcomes in aortic intramural hematoma with an ascending aortic diameter below 50 mm lend support to such a selective strategy, albeit indirect, because intramural hematoma without a discernible entry tear is not identical to overt iatrogenic dissection [27].

Other groups argue that the choice of treatment must be individualized according to the location and extent of the dissection and, above all, the hemodynamic stability of the patient [28]. In this view, conservative management is defensible when the tear is locally confined to the ascending aorta and organ perfusion is not compromised; the strategy consists of strict blood pressure control, serial imaging, and close clinical surveillance [10,21,27].

### 4.4. Our Experience and Thinking

Whenever aortic dissection is suspected during or after a cardiovascular intervention, contrast-enhanced computed tomography angiography of the entire aorta should be obtained as soon as possible, provided that the hemodynamics are stable enough for the patient to leave the interventional suite. In a hemodynamically unstable patient, transport to the CT scanner is itself hazardous, and the operation should proceed directly, with intraoperative TEE used to confirm the diagnosis, localize the intimal tear, and guide the cannulation strategy. This stability-driven diagnostic pathway is consistent with the individualized, hemodynamically anchored management framework advocated in the literature [18,21,28].

Patients developing intervention-related AAD after TAVR or TEER frequently carry a particularly unfavorable baseline risk profile, including advanced age, frailty, severe valvular disease, ventricular dysfunction, and relevant extracardiac comorbidity, all of which can complicate rescue surgery; in our series, patient 1 presented with an ejection fraction of only 28%. This profile characterizes the transcatheter population specifically—spontaneous type A dissection may of course also occur in elderly and comorbid patients, and the two populations overlap rather than differ absolutely. A severely depressed ejection fraction predicts an unstable circulation after cardiopulmonary bypass and difficult weaning, and it materially raises operative risk. Advanced age compounds these difficulties, as the age-related decline in cardiac reserve and in other organ functions impairs tolerance of a prolonged reoperative procedure. In our patients, the extent of reconstruction was dictated primarily by the intraoperative anatomy rather than by age or ventricular function alone. When the root itself is destroyed—entry tear within the sinuses of Valsalva, involvement of the coronary ostia or the annulus, or a destructive root hematoma—replacement with a Bentall procedure addresses all of these lesions and restores valvular competence in a single, reproducible step; it was selected in patients 1, 2, and 4 on these anatomical grounds. In patient 3, in whom the injury to the aortic sinuses remained reconstructible and the dissection involved the arch, the descending thoracic aorta, and all three arch branches, a root-preserving strategy—sinus repair combined with ascending aorta and total arch replacement and a frozen elephant trunk—was performed instead. Conversely, when the arch is not destructively involved and the patient’s overall condition is compromised, a more limited repair without total arch replacement—combined with conservative surveillance of any residual distal hematoma, as in patient 2—can be the wiser compromise.

Another distinctive problem is hemostasis. Antithrombotic regimens after transcatheter valve therapy vary with the intervention and the individual patient; many receive single or dual antiplatelet therapy, and some require long-term anticoagulation. Persistent platelet inhibition, however, may increase the perioperative bleeding risk once an emergency sternotomy is performed: tissues tend to ooze, hemostasis can be difficult to secure, and operative time may lengthen. In the present series, only two patients (patients 1 and 3) had documented dual antiplatelet therapy, whereas the antithrombotic treatment of patients 2 and 4 was not recorded, so no firm conclusion regarding the contribution of this factor can be drawn. The consequences can be grave; insufficient coagulation may progress to pericardial tamponade, prolonging the operation and necessitating additional open procedures to achieve adequate hemostasis. We therefore advocate anticipatory preparation of blood products and platelet concentrate, together with generous allocation of time for meticulous hemostasis before chest closure.

Finally, the arterial return for cardiopulmonary bypass requires forethought. In routine emergencies for spontaneous AAD, femoral artery cannulation is commonly used for rapid establishment of cardiopulmonary bypass, although the cannulation strategy is individualized according to anatomy, hemodynamic status, and institutional expertise. After a transfemoral transcatheter procedure, however, both femoral arteries have usually been instrumented—the access sites may bear punctures, percutaneous closure devices, or local hematomas—and the vessel may no longer be suitable for a large-bore cannula. In this situation, axillary artery cannulation may represent a useful alternative in selected cases, as it provides antegrade arterial perfusion, leaves the femoral access sites undisturbed, and facilitates selective cerebral perfusion should the repair extend into the arch.

## 5. Conclusions

Patients who develop AAD during cardiovascular interventions face numerous challenges and risks, and timely surgical intervention can be life-saving in appropriately selected patients. To ensure surgical success and patient safety, physicians must possess comprehensive understanding of the patient’s condition, develop an individualized surgical plan, closely monitor vital signs and coagulation function intraoperatively, and promptly address potential complications. However, these patients already have cardiac insufficiency or decline in other organ functions, and in addition to receiving significant iatrogenic injuries, they often recover slowly after surgery, leading to longer hospital stays and higher complication rates and mortality. With only four patients, this experience does not establish any preferred operative strategy; it documents the technical feasibility of emergency surgical rescue and distills practical lessons for teams confronting this rare emergency.

## 6. Study Limitations

The findings of this report must be interpreted with caution. Only four patients treated at a single academic center were included, no independent cohort was available to validate our management strategy, and the retrospective, uncontrolled design precludes any firm comparison between treatments. Selection bias is likewise unavoidable in a small case series: the patients who reach the operating room and are reported here represent only a fraction of all those in whom intervention-related aortic injury occurs. The four patients also differed in their index interventions and presumed mechanisms of injury; the series is too small to estimate the incidence of the complication; the procedural mechanism could not be fully characterized in every case; and systematic follow-up imaging was incomplete. Because the condition is so rare, controlled studies are impracticable for any single institution. Collaborative registries and multicenter aggregation of cases therefore represent the only realistic path toward defining optimal surveillance, imaging, and treatment strategies for this devastating complication; until such evidence matures, we offer this experience as a practical reference for teams facing similar emergencies.

## Figures and Tables

**Table 1:** Baseline, procedural, and outcome characteristics of the four patients.

Characteristic	Patient 1	Patient 2	Patient 3	Patient 4
Age (years), sex	83, male	77, female	58, male	72, male
Indication	Severe calcific AS, decompensated HF (NYHA IV)	Severe AS with BAV and 48 mm ascending aneurysm	Severe MR (P2/P3 prolapse, ruptured chordae) with severe TR	Severe calcific AS (BAV, type 0), decompensated HF
Major comorbidities	CKD, hypertension, CAD	Hypertension, CAD	Recent PCI (LAD, 2 DES), pulmonary hypertension	Hypotension, bilateral pleural effusion
LVEF (%)	28	56	54	30
NT-proBNP (pg/mL)	>35,000	207	5113	>35,000
Ascending aorta diameter (mm)	34	48	NR	NR
Antithrombotic therapy before intervention	Aspirin + clopidogrel	NR	Aspirin + clopidogrel (post-PCI)	NR
Intervention (device, access)	TAVR (transfemoral; Evolut PRO 26 mm, Medtronic)	TAVR (right femoral 20-F + left femoral 6-F; Medtronic valve 26 mm exchanged for 29 mm)	TEER (right femoral vein, 24-F guiding sheath; Valveclip-M × 1, A3-P3)	TAVR (Evolut PRO 29 mm, Medtronic)
Timing of injury recognition	During TAVR, after valve deployment	End of TAVR, after final valve release	During clip manipulation	During TAVR, after valve deployment
Clinical presentation	Hypotension with rapidly enlarging pericardial effusion (tamponade)	Hemodynamically stable; intimal detachment on TEE	Hemodynamically stable; widening of ascending aorta on TEE	Circulatory collapse; suspected tamponade
Entry tear	3 mm, below the left coronary ostium (left-noncoronary commissure)	~2 cm transverse laceration of the noncoronary sinus	Not identified in the aorta; atrial septal hematoma with a small perforation at the fossa ovalis	2 mm, below the right coronary ostium, 1 mm above the annulus
Extent of injury	IMH of the noncoronary and left coronary sinuses extending into the ascending aorta	Root dissection; residual arch IMH managed conservatively	Dissection of the ascending aorta, arch, and descending thoracic aorta involving all three arch branches	RVOT hematoma with active bleeding; ascending aortic hematoma
Time to surgery	Same night	Same day (evening)	Next day	Same night
Surgical procedure	TAVR valve explant + Bentall (21 mm mechanical valve/26 mm graft)	TAVR valve explant + Bentall (21 mm mechanical valve/24 mm graft) + CABG × 2 (SVG to RCA and PDA)	Aortic sinus repair + ascending aorta and total arch replacement + FET (CRONUS 26 × 120 mm) + clip removal + MVR (27 mm bioprosthesis)	TAVR valve explant + Bentall (21 mm mechanical valve/24 mm graft)
CPB time (min)	346	NR	419	334
Arterial cannulation	Left femoral artery	Right axillary artery	Right axillary artery	Right axillary artery
Major postoperative complications	Sepsis with severe pneumonia, AKI requiring CRRT, gastrointestinal hemorrhage	Residual arch IMH; pleural effusion (700 mL, drained)	Cardiac arrest (resuscitated), anemia, infection, disturbance of consciousness, hepatorenal dysfunction	Recurrent cardiac arrest, ventricular tachycardia, refractory cardiogenic shock
In-hospital outcome	Discharge against medical advice on POD 18 (ventilator-dependent)	Discharged home on POD 16 with lifelong warfarin	Transferred to cardiology on POD 16; discharged home on POD 24 with no sequelae	Discharge against medical advice on POD 0
Follow-up	Died 2 days after discharge (POD 20)	Clinical: alive and well at 31 months after surgery (September 2026). Imaging: echo at 6 months (August 2024) with normal prosthetic valve and ascending graft function; periodic CT at a local hospital reported unchanged since discharge	Clinical: alive, self-caring and asymptomatic at 42 months (September 2026). Imaging: none after discharge	Discharged against medical advice on the day of surgery on high-dose vasopressors at the family’s request; died immediately after arriving home

AS, aortic stenosis; BAV, bicuspid aortic valve; CABG, coronary artery bypass grafting; CAD, coronary artery disease; CKD, chronic kidney disease; CPB, cardiopulmonary bypass; CRRT, continuous renal replacement therapy; DES, drug-eluting stent; FET, frozen elephant trunk; HF, heart failure; IMH, intramural hematoma; LAD, left anterior descending artery; LVEF, left ventricular ejection fraction; MR, mitral regurgitation; MVR, mitral valve replacement; NR, not recorded; NYHA, New York Heart Association; PCI, percutaneous coronary intervention; POD, postoperative day; PDA, posterior descending artery; RCA, right coronary artery; RVOT, right ventricular outflow tract; SVG, saphenous vein graft; TAVR, transcatheter aortic valve replacement; TEE, transesophageal echocardiography; TEER, transcatheter edge-to-edge repair; TR, tricuspid regurgitation.

**Table 2:** Probable mechanism of aortic injury and predisposing factors for each patient.

Patient	Intervention	Probable Mechanism of Injury	Predisposing Factors
Patient 1	TAVR	Balloon-valve interaction and prosthesis deployment trauma at the left-noncoronary commissure, producing a 3 mm intimal tear below the left coronary ostium	Heavy calcification of the aortic root, diffuse atherosclerosis
Patient 2	TAVR	Radial force of the prosthesis: a 26 mm valve with suboptimal anchoring was exchanged for a 29 mm valve, producing a transverse laceration of the noncoronary sinus	Bicuspid aortic valve, pre-existing 48 mm ascending aortic aneurysm, valve up-sizing
Patient 3	TEER	Possible transseptal puncture- or guiding-sheath-related atrial septal injury (hematoma with a small perforation at the fossa ovalis) with presumed propagation to the aortic root and ascending aorta; no aortic entry tear identified intraoperatively	Severe pulmonary hypertension, four-chamber enlargement, recent PCI on dual antiplatelet therapy
Patient 4	TAVR	Prosthesis deployment trauma in a heavily calcified bicuspid (type 0) root, producing a 2 mm tear just below the right coronary ostium	Heavy calcification, bicuspid aortic valve (type 0), severely reduced LVEF

## Data Availability

Data will be made available on request.

## Supplementary Materials

The following supporting information can be downloaded at https://www.mdpi.com/article/10.3390/jcm15207901/s1. The CARE Checklist of this study.

## Abbreviations

AAD	acute type A aortic dissection
CTA	computed tomographic angiography
TAVR	transcatheter aortic valve replacement
TEER	transcatheter edge-to-edge repair
TEE	transesophageal echocardiography
TEVAR	thoracic endovascular aortic repair
