# Supplementary material for: Experiences in the Management of Type A Aortic Dissection Following Cardiovascular Interventions: A Case Series Report
Source: J Clin Med. 2026 Oct 13;15(20):7901. doi: 10.3390/jcm15207901 (PMC13705728; doi:10.3390/jcm15207901)
Supplement: Supplementary file 1 [file jcm-15-07901-s001.zip › jcm-4571910-supplementary.pdf]

CARE Checklist

Manuscript: Experiences in the Management of Type A Aortic Dissection Following Cardiovascular Interventions: A Case Series Report

Manuscript ID: jcm-4571910 (Journal of Clinical Medicine) — Revised version, 2026

Note: Page numbers refer to the clean copy of the revised manuscript file.

Topic	Item	Checklist item description	Reported on (Page/Section)
Title	1	The diagnosis or intervention of primary focus followed by the words "case report"	Page 1, Title. The revised title now ends with "A Case Series Report".
Key Words	2	2 to 5 key words that identify diagnoses or interventions in this case report, including "case report"	Page 1, Keywords. "case report" has been added to the keyword list.
Abstract	3a	Introduction: What is unique about this case and what does it add to the scientific literature?	Page 1, Abstract (sentences 1–2).
Abstract	3b	Main symptoms and/or important clinical findings	Page 1, Abstract (sentence 3: four patients with AAD after TAVR/TEER).
Abstract	3c	The main diagnoses, therapeutic interventions, and outcomes	Page 1, Abstract (sentences 4–6: Bentall procedure × 3, each requiring transcatheter valve explantation, one with concomitant coronary artery bypass grafting; aortic sinus repair with ascending aorta and total arch replacement using a frozen elephant trunk, with removal of the TEER clip and mitral valve replacement × 1; one recovered and discharged home, one transferred to cardiology, two discharged against medical advice).
Abstract	3d	Conclusion—What is the main "take-away" lesson(s) from this case?	Page 1, Abstract (final sentences: emergency surgical rescue can be life-saving; the Bentall procedure proved feasible in this selected cohort; individualized planning essential).
Introduction	4	One or two paragraphs summarizing why this case is	Pages 1–2, Introduction (four paragraphs, refs 1–10).

		unique (may include references)	
Patient Information	5a	De-identified patient specific information	Pages 3–4, Case Presentations (four patients: 83-year-old male; 77-year-old female; 58-year-old male; 72-year-old male).
Patient Information	5b	Primary concerns and symptoms of the patient	Pages 3–4, Case Presentations (chest tightness, dyspnea, heart failure symptoms).
Patient Information	5c	Medical, family, and psycho-social history including relevant genetic information	Pages 3–4, Case Presentations (aortic stenosis, mitral regurgitation, comorbidities; no family/genetic syndromes identified).
Patient Information	5d	Relevant past interventions with outcomes	Pages 3–4, Case Presentations (prior TAVR × 3, TEER × 1 with periprocedural dissection).
Clinical Findings	6	Describe significant physical examination (PE) and important clinical findings	Pages 3–4, Case Presentations (hemodynamic status, pericardial tamponade signs); Page 5, Results.
Timeline	7	Historical and current information from this episode of care organized as a timeline	Pages 3–5, Case Presentations → Treatments → Results (each patient described chronologically: admission → intervention → dissection → emergency surgery → outcome).
Diagnostic Assessment	8a	Diagnostic testing (such as PE, laboratory testing, imaging, surveys)	Pages 3–4 (echocardiography, CTA, NT-proBNP, ejection fraction); Page 7, Our experience (CTA/TEE strategy).
Diagnostic Assessment	8b	Diagnostic challenges (such as access to testing, financial, or cultural)	Page 7, Our experience (hemodynamically unstable patients cannot be transferred for CT; intraoperative TEE).
Diagnostic Assessment	8c	Diagnosis (including other diagnoses considered)	Pages 3–5 (AAD confirmed by CTA and intraoperative findings; differential considerations in Discussion).
Diagnostic Assessment	8d	Prognosis (such as staging in oncology) where applicable	Page 5, Discussion (30-day mortality ≈67% of intervention-related AAD, contextualized from a published cohort); Page 5, Results (individual outcomes).
Therapeutic Intervention	9a	Types of therapeutic intervention (such as pharmacologic, surgical, preventive, self-care)	Page 4, Treatments (emergency surgery: Bentall procedure × 3, with transcatheter valve explantation in all three and concomitant coronary artery bypass grafting in one; aortic

			sinus repair with ascending aorta and total arch replacement using a frozen elephant trunk (Sun's procedure) × 1).
Therapeutic Intervention	9b	Administration of therapeutic intervention (such as dosage, strength, duration)	Page 4, Treatments (emergency open-heart surgery with cardiopulmonary bypass; cannulation strategy discussed on Page 6).
Therapeutic Intervention	9c	Changes in therapeutic intervention (with rationale)	Page 4, Treatments (e.g., urgent transcatheter valve explantation before Bentall repair in patient 2); Page 7, Our experience (limited vs. total arch repair).
Follow-up and Outcomes	10a	Clinician and patient-assessed outcomes (if available)	Page 5, Results (each outcome now reported explicitly: patient 4 died immediately after arriving home following discharge against medical advice on the day of surgery; patient 1 died 2 days after discharge; patient 2 and patient 3 survived to discharge); Table 1, Follow-up row (telephone follow-up, September 2026).
Follow-up and Outcomes	10b	Important follow-up diagnostic and other test results	Page 5, Results; postoperative CTA (Figures 6–7).
Follow-up and Outcomes	10c	Intervention adherence and tolerability (How was this assessed?)	Not applicable—single emergency surgical episode per patient, no long-term adherence assessment.
Follow-up and Outcomes	10d	Adverse and unanticipated events	Page 5, Results (patient 1 discharged against medical advice on postoperative day 18 while critically ill and ventilator-dependent, died 2 days after discharge; patient 4 discharged on the day of surgery in refractory cardiogenic shock and died after arriving home); intraoperative findings (pericardial tamponade, active bleeding).
Discussion	11a	A scientific discussion of the strengths AND limitations associated with this case report	Page 9, Study Limitations (single center, four patients, no validation cohort; incomplete post-discharge imaging follow-up; future directions).

Discussion	11b	Discussion of the relevant medical literature with references	Pages 5–8, Discussion (refs 12–28: incidence, mechanisms, management strategies, outcomes).
Discussion	11c	The scientific rationale for any conclusions (including assessment of possible causes)	Pages 5–8 (mechanisms of injury, patient substrate, treatment rationale for Bentall vs. limited repair).
Discussion	11d	The primary “take-away” lessons of this case report (without references) in a one paragraph conclusion	Page 8, Conclusions.
Patient Perspective	12	The patient should share their perspective in one to two paragraphs on the treatment(s) they received	Not applicable—retrospective case series of four emergency patients (two deaths: patient 4, discharged against medical advice on the day of surgery, died immediately after arriving home, and patient 1 died 2 days after discharge); patient narrative not obtainable for this report.
Informed Consent	13	Did the patient give informed consent? Please provide if requested	Yes. Page 9, Ethics Statement: written informed consent for publication of clinical details and images was obtained from the patients or their legal representatives.
